# Experience in setting up non-robotic minimally invasive direct coronary artery bypass grafting in a non-routine off-pump coronary artery bypass center

**DOI:** 10.1186/s40001-025-02320-0

**Published:** 2025-01-31

**Authors:** Yukiharu Sugimura, Tomoyuki Suzuki, Sebastian Johannes Bauer, Friederike Irmgard Schoettler, Moritz Benjamin Immohr, Michael André Maliwa, Arash Mehdiani, Lachmandath Tewarie, Gereon Schaelte, Ajay Moza, Payam Akhyari

**Affiliations:** 1 Department of Thoracic and Cardiovascular Surgery, West-German Heart and Vascular Center Essen, University Duisburg-Essen, Essen, Germany; 2 Department of Cardiac Surgery, Medical Faculty and RWTH University Hospital Aachen, RWTH Aachen University, Aachen, Germany; 3 Department of Anesthesiology, Medical Faculty and RWTH University Hospital Aachen, RWTH Aachen University, Aachen, Germany; 4 Department of Anesthesiology, Intensive Care and Emergency Medicine, Hermann-Josef General Hospital Erkelenz, Erkelenz, Germany

**Keywords:** Minimally invasive direct coronary artery bypass, MIDCAB, MICS-CABG, Hybrid

## Abstract

**Background:**

The safety of minimally invasive direct coronary artery bypass (MIDCAB) has been proven. Nevertheless, reports on clinical outcomes in MIDCAB and the learning curve of this challenging technique in a non-routine off-pump coronary artery bypass (OPCAB) center are still limited. Here, we introduce our clinical outcomes of non-robotic MIDCAB.

**Methods:**

Between August 2022 and March 2024, 72 consecutive patients with a mean age of 67.4 ± 9.5 years underwent non-robotic MIDCAB (defined as off-pump bypass grafting of the left internal mammary artery to the left anterior descending artery through left-sided mini-thoracotomy). We analyzed operation time and incidence of major adverse cardiac and cerebrovascular events (MACCE). Further, subgroup analyses included body mass index (BMI) with a cut-off of 30 kg/m^2^ [BMI ≧ 30: *n* = 18 (25.0%)] and body surface area (BSA) with a cut-off of 2.0 m^2^ [BSA ≧ 2.0: *n* = 34 (47.2%)].

**Results:**

All patients survived, whereas MACCE occurred in 4 patients (5.6%). By correlation analyses, no learning curve for operation time was observed in all cases analysis (*p* = 0.79), but MACCE (*n* = 4, 5.6%) exclusively observed in the first 34 patients. Furthermore, BMI ≧ 30 or BSA ≧ 2.0 was not significantly related to longer operation time (*p* = 0.42 and *p* = 0.52, respectively) and MACCE (*p* = 0.26 and *p* = 0.35, respectively). In addition, body size had no effect on operation time according to multiple regression analysis (*p* = 0.36).

**Conclusions:**

Our study suggested that implementing non-robotic MIDCAB can be safely accomplished at a center with no previous routine in OPCAB surgery, even for patients with bigger body sizes. MACCE occurs more frequently in the early stages when adopting this surgical technique.

**Supplementary Information:**

The online version contains supplementary material available at 10.1186/s40001-025-02320-0.

## Background

In recent years, minimally invasive cardiac surgery (MICS) techniques have become established in cardiac surgery due to advances in surgical instruments and medical materials [1, 2]. Minimally invasive direct coronary artery bypass grafting (MIDCAB) or minimally invasive coronary artery bypass grafting (CABG) through a limited thoracotomy approach is increasingly used as a routine procedure because it is superior to conventional sternotomy in terms of esthetics, lower risk of bleeding and wound infection, and faster recovery [3–5]. In the 2018 European Society of Cardiology/European Association of Cardiothoracic Surgery (ESC/EACTS) guidelines, minimally invasive CABG through a limited thoracotomy approach was listed for the first time and accordingly should be considered for patients with isolated left anterior descending artery (LAD) stenosis or as part of hybrid procedures (class IIa recommendation, level of evidence B) [6]. However, performing minimally invasive CABG via mini-thoracotomy requires sufficient experience with off-pump CABG (OPCAB) in a surgical team and overcoming the difficulty of assisting other personnel with the surgical procedure due to the limited surgical view. In Germany, the rate of OPCAB was only 26.2% of all isolated CABG patients in 2023 (28,996 patients) [7]. Therefore, minimally invasive CABG via a limited thoracotomy approach is still an evolving procedure and is not performed in many cardiac centers, so global standards for the safe implementation of minimally invasive CABG, especially in non-routine off-pump bypass centers, have not yet been discussed.

The university hospital RWTH Aachen is one of the leading cardiac centers in Germany. However, OPCAB procedures, including MIDCAB, have not been routinely performed in the past. Between August 2021 and July 2022, only 4.7% (*n* = 24) of all isolated CABG patients (*n* = 503) underwent OPCAB at this center. More than half of these OPCAB patients received a single bypass (left internal mammary artery (LIMA) to LAD) via full sternotomy. It means that MIDCAB was avoided in many patients due to various reasons, such as risk considerations and presumably also due to the lack of routine, especially in large patients. Since August 2022, with the appointment of new team members, individually equipped with solid experience in OPCAB as well as MIDCAB (≥20 cases/year) in their previous hospital, the team has decided to start an initiative to implement a routine MIDCAB program for all-comer patients fulfilling the criteria for MIDCAB according to current guidelines [8, 9]. Challenges were expected with respect to interdisciplinary and interprofessional management of patients during MIDCAB, as well as with regard to dissemination of the expertise within the surgical team. Here, we would like to share our first experiences of implementing MIDCAB in a previous non-routine OPCAB center.

## Methods

### Study design and data collection

**Fig. 1:**
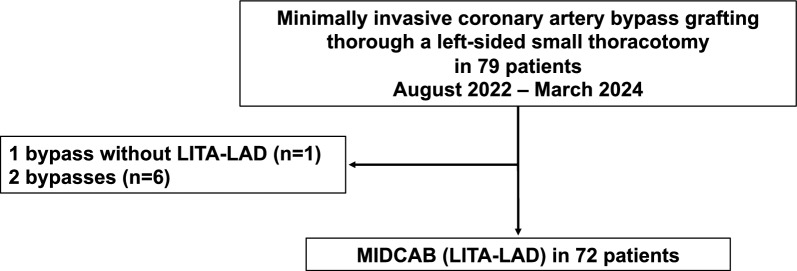
The CONSORT diagram depicting study population. Seventy-nine consecutive patients underwent minimally invasive CABG, of whom 72 patients underwent non-robotic MIDCAB with single bypass made of LIMA to LAD. CABG: coronary artery bypass grafting; LAD: left anterior descending artery; LIMA: left internal mammary artery; MIDCAB: minimally invasive direct coronary artery bypass

Between August 2022 and March 2024, 79 consecutive patients underwent minimally invasive CABG (defined as OPCAB through left-sided mini-thoracotomy) in our department, of whom 72 patients underwent non-robotic MIDCAB (defined as minimally invasive CABG with LIMA to LAD) (Fig. 1). On the other hand, 6 further patients underwent minimally invasive CABG with 2 bypasses and the remaining 1 patient underwent 1 saphenous vein bypass on the right coronary artery. In the latter 7 patients there was no in-hospital mortality, no conversion to full sternotomy, and moreover no major adverse cardiac and cerebrovascular events (MACCE). These 7 patients were excluded from further analysis to obtain a homogenous group receiving classic MIDCAB, i.e., LIMA to LAD in off-pump technique via mini-thoracotomy. The remaining 72 patients were analyzed in this study, investigating perioperative patient status, clinical outcomes including operative time and incidence of MACCE (defined as all-cause death, stroke, bypass-related myocardial infarction, followed by revascularization). Subgroup analyses included obese patients as defined by body mass index (BMI) ≧ 30 [*n* = 18 (25.0%)] or large patients as defined by body surface area (BSA) ≧ 2.0 [*n* = 34 (47.2%)]. The primary endpoint of the study was in-hospital mortality. The secondary endpoint of the study was MACCE and operation time. In this single-center retrospective study, all preoperative characteristics, perioperative clinical information and postoperative outcomes were prospectively entered into the hospital data management and quality assurance system and retrospectively retrieved for study purposes.

### Indication, surgical procedure of MIDCAB

During the study period, in principle all patients with single-vessel disease of the LAD were considered as candidates for MIDCAB. The only strict contraindication to MIDCAB at our center was a history of previous left-sided thoracotomy. Other risk factors for conversion to full sternotomy, such as a history of prior radiotherapy or severe chronic obstructive pulmonary disease (COPD), were considered as relative contraindications to this procedure. Patients were individually assessed by the surgical team to confirm the indication for MIDCAB. According to the current ESC/EACTS guidelines, the indication for MIDCAB as part of a hybrid procedure in high-risk patients was determined by the interdisciplinary heart team [8, 9].

A total of 5 surgeons performed MIDCAB on 72 patients. Three of them were new surgeons with sufficient experience (≥20/year). They operated on a total of 60 patients. In the remaining 12 patients, 2 other surgeons performed MIDCAB under the supervision of the new chief surgeon. Our standard MIDCAB procedure is performed via a left mini-thoracotomy through the fourth or fifth intercostal space in a supine position with slightly elevated left lateral chest. In keeping with our therapeutic vision of ensuring the same quality of operation in minimally invasive access as with full sternotomy, even in the setting of MICS, the LIMA is harvested in a fully skeletonized fashion proximal to the identification of the left subclavian vein in all patients. After effective anticoagulation with heparin, end-to-side anastomosis of the LIMA to the LAD is performed using a continuous suture and a stabilizer system as known for OPCAB. We routinely place an intracoronary shunt. Transit time flow measurement (TTFM) is then performed in all patients. If the result of the TTFM is not satisfactory, e.g., mean graft flow < 20 ml/min and/or pulsatility index (PI) > 5.0, revision of the bypass is considered. If only PI was not lower than 5.0, we temporarily occluded the proximal LAD, then TTFM was performed again to evaluate the competitive flow from the native LAD. If only the graft flow was low, we used the papaverine to the adventitia of the LITA (not inject in the LITA) and kept the systemic blood pressure higher, waited a time in the operating room, and then we reevaluated the graft flow. If intraoperative echocardiographic or electrocardiographic findings suggested ischemia, all anastomoses were revised.

### Postoperative graft assessment

At our institute, the patency of the bypass grafts was not assessed in all patients routinely. The postoperative coronary angiography (CAG) was done only in patients with (1) newly abnormal electrocardiograms, and/or (2) the elevation of postoperative myocardial enzymes on regular check-ups every 4 h, and/or (3) hemodynamic instability.

### Statistical analysis

Statistical analyses were performed using SPSS Statistics version 28.0 (IBM Corp., Armonk, N.Y., USA). Descriptive and comparative (*χ*^2^-test, Mann–Whitney *U*-test) statistics were calculated using this program. However, Fisher's exact test was used instead of the *χ*^2^-test for a minimum expected value of less than five. The data of the interval-scaled variables were expressed as mean ± standard deviation. Regarding a correlation analysis, Spearman’s test was administered in this study. Multiple regression analysis was used to examine the several factors that influenced the outcome. Statistical significance was set at *p* < 0.05. Statistical significance was set at *p* < 0.05.

## Results

### Baseline clinical characteristics

Baseline clinical characteristics are shown in Table 1. Most of our patients were male (86.1%) and the mean age of all patients was 67.4 ± 9.5 years. The mean BMI and BSA were 27.6 ± 3.9 kg/m^2^ and 2.0 ± 0.2 m^2^, respectively. Left ventricular ejection fraction (53.3 ± 10.4%) and Euro Score II (1.5 ± 1.2) were unremarkable in our patients. In contrast, over 75% of all patients had class 2 or greater Canadian Cardiovascular Society and/or New York Heart Association status. In addition, a relevant proportion of patients had central or peripheral vascular disease (*n* = 13, 18.1%) and COPD (*n* = 11, 15.3%). A total of 21 patients (29.2%) underwent MIDCAB planned as a hybrid procedure with either percutaneous coronary intervention (PCI) (*n* = 18, 85.7%) or transcatheter aortic valve implantation (TAVI) (*n* = 3, 14.3%) already planned at the time of MIDCAB surgery. Further information on patients undergoing MIDCAB as a hybrid procedure is presented in Suppl. Table 1.

**Table 1:** Baseline clinical characteristics

	Patients (*n* = 72)
Age (year)	67.4 ± 9.5
Male, *n* (%)	62 (86.1)
BMI (kg/m^2^)	27.6 ± 3.9
≥ 30	18 (25.0)
BSA (m^2^)	2.0 ± 0.2
≥ 2	34 (47.2)
CCS classification ≥ 2, *n* (%)	54 (75.0)
NYHA classification ≥ 2, *n* (%)	55 (76.4)
One vessel disease	54 (75.0)
Two or three vessel disease	18 (25.0)
Euro Score II	1.0 (0.7–1.7)
Arterial hypertension, *n* (%)	51 (70.8)
Hyperlipidemia, *n* (%)	31 (43.1)
Diabetes, *n* (%)	21 (29.2)
Central or peripheral vascular disease, *n* (%)	13 (18.1)
COPD, *n* (%)	11 (15.3)
History of PCI, *n* (%)	8 (11.1)
LVEF (%)	53.3 ± 10.4
Planned as hybrid procedures, *n* (%)	21 (29.2)
PCI	18 (85.7)^a^
TAVI	3 (14.3)^a^

Data documented as n (%) or mean ± standard deviation or median (1. quartile-3. quartile)

BMI: body mass index; BSA: body surface area; CCS: Canadian Cardiovascular Society; COPD: chronic obstructive pulmonary disease; NYHA: New York Heart Association; LVEF: left ventricular ejection fraction; PCI: percutaneous coronary intervention; TAVI: transcatheter aortic valve implantation;

^a^% among 21 patients as hybrid procedures

### Clinical outcomes of MIDCAB

The clinical outcomes of MIDCAB are presented in Table 2. The in-hospital mortality rate was 0%. The mean operative time for all patients was 188 ± 46 min, ranging from 125 to 406 min. More than half of our patients (59.7%) were extubated in the operating room, with only one patient (1.4%) requiring reintubation. Intraoperative conversion to full sternotomy was performed in 4 patients (5.6%). Two patients had severe adhesions in situ, while a local injury or dissection of the LIMA was the cause of conversion to full sternotomy in the remaining two patients. Postoperative early graft failure (EGF) was observed in 3 patients requiring re-bypass surgery. The incidence of MACCE was 5.6% (*n* = 4), including the aforementioned 3 patients with EGF and one patient with postoperative prolonged reversible ischemic-neurological deficit who was free of clinical symptoms at discharge. Of note, surgical site infection (SSI) was found in 6 patients (8.3%), including 4 male patients (6.5% of all male patients) and 2 female patients (20% of all female patients). No patients scheduled for hybrid procedures required unplanned postoperative PCI or TAVI. This means that PCI or TAVI could be performed electively after recovery from the index MIDCAB procedure in all “hybrid” patients.

**Table 2:** Clinical outcomes of MIDCAB

	Patients (*n* = 72)
Operation time (min)	187.7 ± 46.2
Conversion to sternotomy, *n* (%)	4 (5.6)
Conversion to CPB, *n* (%)	0 (0.0)
Extubation on table, *n* (%)	43 (59.7)
Re-intubation, *n* (%)	1 (1.4)
ICU stay (h)	23.0 (18.8–27.3)
Hospital stay (d)	6.9 ± 3.8
Transfusion of red cell concentrates (units)	0 (0.0–0.0)
Transfusion of red cell concentrates, *n* (%)	8 (11.1)
In-hospital mortality, *n* (%)	0 (0.0)
MACCE, *n* (%)	4 (5.6)
Postoperative early graft failure, *n* (%)	3 (4.2)
Re-bypass operation, *n* (%)	3 (4.2)
PCI, *n* (%)	0 (0.0)
SSI, *n* (%)	6 (8.3)
New onset of postoperative dialysis, *n* (%)	1 (1.4)

Data documented as *n* (%) or mean ± standard deviation or median (1. quartile-3. quartile)

CPB: cardiopulmonary bypass; ICU: intensive care unit; MACCE: major adverse cardiac and cerebrovascular events; MIDCAB: minimally invasive direct coronary artery bypass grafting; PCI: percutaneous coronary intervention; SSI: surgical site infection

### Learning curve evaluation

**Fig. 2:**
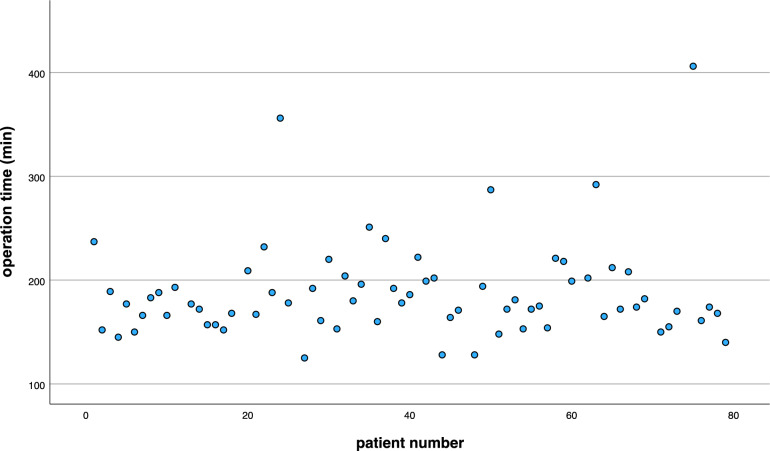
The correlation analysis between operative time and the number of patients. The correlation analysis revealed no learning curve for operative time (*p* = 0.79)

Figure 2 shows the correlation analysis between operative time along the consecutive series of patients analyzed. There was no learning curve for operative time (*p* = 0.79). Regarding the occurrence of MACCE, however, the last MACCE occurred in the 34th patient in the descriptive analysis (Suppl. Figure 1).

### Clinical outcomes according to patients' physiques

**Fig. 3:**
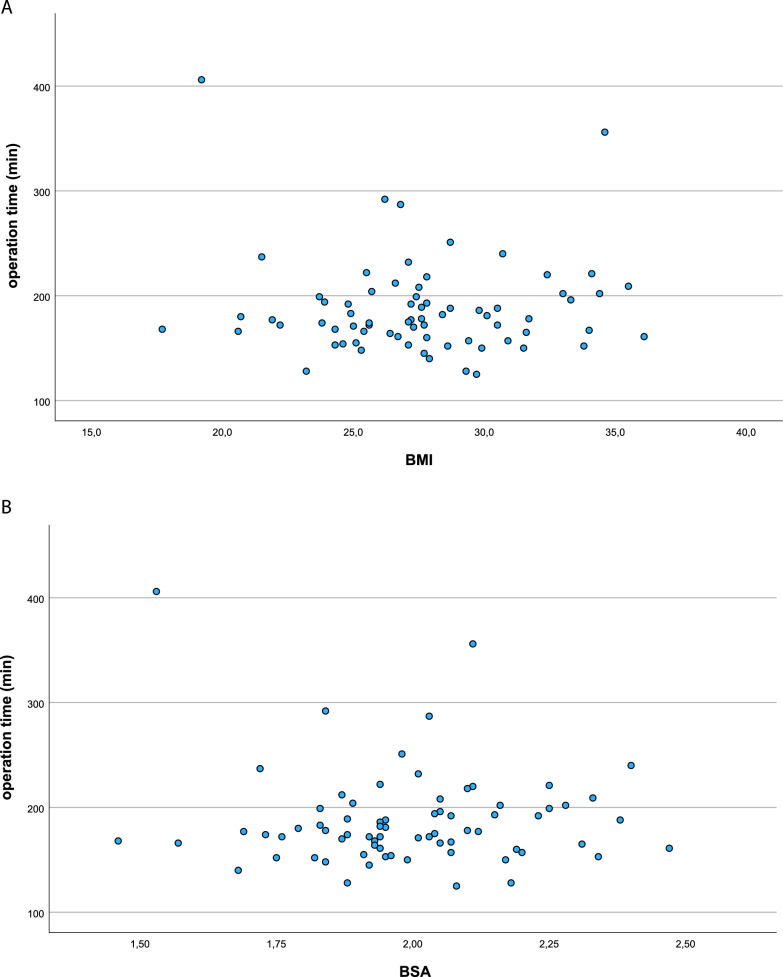
The correlation analysis between operation time and patients’ body size. No correlation was found between operation time and **A** BMI (*p* = 0.79) or **B** BSA (*p* = 0.39). BMI, body mass index (kg/m^2^); BSA: body surface area (m^2^)

Table 3 shows the comparative analysis of operation time and MACCE according to patients' physiques. BMI ≧ 30 kg/m^2^ or BSA ≧ 2.0 m^2^ was not significantly related to longer operation time (*p* = 0.42 and *p* = 0.52, respectively) and MACCE (*p* = 0.26 and *p* = 0.35, respectively) in this cohort. Further, there was no correlation between operation time and patients’ body size (BMI: *p* = 0.79, BSA: *p* = 0.39) (Fig. 3A and B). Furthermore, we did not find an effect of body size, i.e., BMI and BSA, on operation time according to the result of multiple regression analysis (*p* = 0.36).

**Table 3:** Comparing clinical outcomes according to patients' physiques

	BMI < 30 kg/m^2^ (*n* = 54)	BMI ≥ 30 kg/m^2^ (*n* = 18)	p	BSA < 2 m^2^ (n = 38)	BSA ≥ 2 m^2^ (*n* = 34)	p
Operation time (min)	185.2 ± 45.8	195.4 ± 47.8	0.4	184.3 ± 48.7	191.2 ± 43.6	0.5
MACCE, *n* (%)	2 (3.7)	2 (11.1)	0.3	3 (7.9)	1 (2.9)	0.4

Data documented as *n* (%) or mean ± standard deviation

BMI: body mass index; BSA: body surface area; MACCE: major adverse cardiac and cerebrovascular events

## Discussion

Hybrid coronary revascularization and MIDCAB as a component of hybrid procedures, e.g., in combination with TAVI, have gained increasing interest in the cardiovascular community. This study explores the safety of implementing MIDCAB for all-comer patients in an institutional environment not routinely performing OPCAB and minimally invasive CABG. The main findings of this study are (1) safe performance of MIDCAB without in-hospital mortality; (2) sporadic conversion to full sternotomy mainly due to LIMA problems (*n* = 2, 2.7%); (3) absence of a remarkable learning curve with regard to operation time, but MACCE (*n* = 4, 5.6%) exclusively observed in the first 34 patients; (4) successful completion of the pre-specified hybrid concept without emergency interventions in all patients; (5) no significant differences for clinical outcomes according to patient physique; (6) relatively high incidence of SSI in the whole cohort (*n* = 6, 8.3%), especially in female patients (*n* = 2, 20% of all female patients).

In recent decades, the choice of interventional or surgical therapy for coronary revascularization in patients with single-vessel disease, so-called LAD disease, has been well argued. Published data on OPCAB through full sternotomy versus MIDCAB for LIMA-LAD bypass suggest a comparable early and late mortality rate. In a pooled analysis of studies spanning nearly 2 decades, MIDCAB was associated with an increased risk of significant early complications, but resulted in a shorter hospital stay [10]. Notably, most recent studies have suggested a very low incidence of significant early complications [11–13]. Moreover, the long-term results of MIDCAB up to 20 years have shown excellent clinical outcomes with evidence of comparable expected survival in the matched reference population [14]. MIDCAB has also been shown to result in lower all-cause mortality at long-term follow-up as compared to PCI for proximal LAD lesions [15]. The present study adds to pre-existing body of evidence by demonstrating similar MACCE outcomes and comparable operative times in patients with large body size. These findings may be expected to support MIDCAB procedure, which is once established at an institution, will most likely become the first-choice procedure for patients with significant LAD disease.

However, reports focusing on the safe implementation of non-robotic MIDCAB are still scarce. We found that a comprehensive PubMed search using the keywords “MIDCAB” and “implementation” yielded only a few publications (*n* = 6, retrieved on 10th October 2024). More importantly, there are no reports analyzing the critical implementation period, when non-robotic MIDCAB is introduced to a surgical center with no previous routine in OPCAB. For a safe MIDCAB, as well as for a minimally invasive CABG procedure, we believe that the surgical team needs sufficient experience in OPCAB to be familiar with appropriate heart positioning while maintaining patient's hemodynamics. Moreover, previous OPCAB experience aids in becoming familiar with OPCAB instruments. Regarding the quality of OPCAB performance, a recent multicenter retrospective study describes that surgeon experience and center volume may play an essential role for improving early outcomes after OPCAB [16]. Considering the limited surgical space, which restricts the work of the surgeon's assistants and even the visualization of the surgical field for other medical staff in the setting of the minimally invasive approach, level of experience in OPCAB procedure for the surgical team would be more essential for the safe implementation of the minimally invasive CABG. Although the center volume of OPCAB and MIDCAB procedures at our university hospital was low, implementation of MIDCAB was achieved safely without any in-hospital mortality.

Nevertheless, considering the operation time and the incidence of MACCE, our clinical results were slightly different than other recent reports [11–13]. Regarding operation time, harvesting method of LIMA may be a relevant factor. We perform a skeletonized method for LIMA harvest extending proximally up to the left subclavian vein, with endoscopic guidance in case of difficult anatomical setting. In such setting we observed LIMA injuries in 2 patients (2.7%). In a previous report focusing on LIMA harvesting method and comparing full sternotomy vs. non-robotic MIDCAB vs. robotic MIDCAB, no LIMA injuries were found in the full sternotomy group with a mean harvesting time of 36.9 ± 14.3 min, whereas the incidence of LIMA injuries was 1.35% in the full MIDCAB groups with a mean "non-robotic" harvesting time of 74.4 ± 24.2 min [17]. Therefore, we believe that the first step to a successful MIDCAB procedure is safe LIMA harvesting and that more attention should be granted to LIMA harvesting at the beginning of MIDCAB. Although we found no correlation between growing experience (as reflected by the number of individual patients in consecutive series), the last MACCE occurred in the 34th patient, suggesting that there may be a learning curve resulting in better clinical outcomes for MIDCAB [18]. These findings support the hypothesis that pre-existing experience of OPCAB procedure within the surgical team promotes safe implementation of MIDCAB.

Although the benefits of MIDCAB have been well reported, we should pay more attention to the high incidence of SSI. Other reports have similarly documented that the incidence of SSI is higher in MIDCAB (4.7–37%) than in OPCAB utilizing full sternotomy (0–13%) [10]. We regard the rate of SSI in our cohort as relevant and subject to future efforts for optimization, especially in female patients. Cautiously, this means that a strategy to prevent SSI should be given more consideration in all MIDCAB patients.

This study has several limitations. First, as a single-center retrospective study, the cohort size was limited and typical bias inherent to single-center studies cannot be excluded. Second, postoperative CAG was not routinely performed in all patients at our institution. Therefore, it might not be sufficient to conclude on the quality of the graft. Thirdly, MIDCAB was performed by 5 surgeons in this study. This could introduce a kind of operator bias due to the heterogeneity of the data we obtained. In addition, we did not analyze clinical outcome parameters during the further follow-up period. From another perspective, 21 patients (29.2%) in our study underwent MIDCAB as a hybrid procedure. In this sense, our patient group was somewhat heterogeneous to analyze. In addition, the evidence on hybrid procedures is still insufficient for solid statements on the value of such approach, despite recently published data [19, 20].

With these limitations on mind, our study aims to report our first experience of the safe implementation of MIDCAB in an institution with no previous routine in OPCAB. We believe that our study provides novel insights of interest for surgeons planning to initiate or disseminate MIDCAB activity to a new center, especially at institutions without enough prior OPCAB experience.

## Conclusion

Our study suggests that introducing non-robotic MIDCAB is safe even for larger patients in a center with no routine OPCAB experience. Despite the lack of identification of a learning curve for operative time, this study suggests that MACCE occurs more frequently in the early stages when adopting this surgical technique.

## Supplementary Information

Supplementary Material 1: Suppl. Figure 1. The graphical explanation of the development of MIDCAB patients and the occurrence of MACCE. The number of MIDCAB patients has gradually increased. MACCE occurred in 4 patients with the last MACCE on the 34th patient. MACCE, major adverse cardiac and cerebrovascular event; MIDCAB, minimally invasive direct coronary artery bypass.

Supplementary Material 2: Suppl. Table 1. Representative information in MIDCAB patients as a hybrid procedure.

## Data Availability

No datasets were generated or analysed during the current study.

## References

[1] Lapierre H, Chan V, Sohmer B, Mesana TG, Ruel M. Minimally invasive coronary artery bypass grafting via a small thoracotomy versus off-pump: a case-matched study. Eur J Cardiothorac Surg. 2011;40(4):804–10. 21393011 10.1016/j.ejcts.2011.01.066

[2] Groh MA, Sutherland SE, Burton HG 3rd, Johnson AM, Ely SW. Port-access coronary artery bypass grafting: technique and comparative results. Ann Thorac Surg. 1999;68(4):1506–8. 10543555 10.1016/s0003-4975(99)00949-2

[3] Yang D, Zhang K, Li J, Wei D, Ma J, Wang Y, et al. Ninety-seven cases of experiences with the left thoracotomy approach for off-pump conventional revascularization: a retrospective cohort study. J Thorac Dis. 2022;14(10):3915–23. 36389332 10.21037/jtd-22-1162 PMC9641355

[4] Rufa M, Ursulescu A, Nagib R, Albert M, Franke UFW. Hybrid total arterial minimally invasive off-pump coronary revascularization and percutaneous coronary intervention strategy for multivessel coronary artery disease: a cohort study with a median 11-year follow-up. Cardiovasc Diagn Ther. 2024;14(2):272–82. 38716312 10.21037/cdt-23-413 PMC11070998

[5] Ushioda R, Hirofuji A, Yoongtong D, Sakboon B, Cheewinmethasiri J, Lokeskrawee T, et al. Multi-vessel coronary artery grafting: analyzing the minimally invasive approach and its safety. Front Cardiovasc Med. 2024;11:1391881. 38774658 10.3389/fcvm.2024.1391881 PMC11106462

[6] Neumann FJ, Sousa-Uva M, Ahlsson A, Alfonso F, Banning AP, Benedetto U, et al. 2018 ESC/EACTS Guidelines on myocardial revascularization. Eur Heart J. 2019;40(2):87–165. 30165437 10.1093/eurheartj/ehy394

[7] Beckmann A, Meyer R, Eberhardt J, Gummert J, Falk V. German heart surgery report 2023: the annual updated registry of the German Society for Thoracic and Cardiovascular Surgery. Thorac Cardiovasc Surg. 2024;72(5):329–45. 39079552 10.1055/s-0044-1787853

[8] Byrne RA, Rossello X, Coughlan JJ, Barbato E, Berry C, Chieffo A, et al. 2023 ESC Guidelines for the management of acute coronary syndromes. Eur Heart J. 2023;44(38):3720–826. 37622654 10.1093/eurheartj/ehad191

[9] Vrints C, Andreotti F, Koskinas KC, Rossello X, Adamo M, Ainslie J, et al. 2024 ESC Guidelines for the management of chronic coronary syndromes. Eur Heart J. 2024;45(36):3415–537. 39210710 10.1093/eurheartj/ehae177

[10] Florisson DS, DeBono JA, Davies RA, Newcomb AE. Does minimally invasive coronary artery bypass improve outcomes compared to off-pump coronary bypass via sternotomy in patients undergoing coronary artery bypass grafting? Interact Cardiovasc Thorac Surg. 2018;27(3):357–64. 29579209 10.1093/icvts/ivy071

[11] Monsefi N, Alaj E, Sirat S, Bakhtiary F. Postoperative results of minimally invasive direct coronary artery bypass procedure in 234 patients. Front Cardiovasc Med. 2022;9:1051105. 36704468 10.3389/fcvm.2022.1051105 PMC9871774

[12] Weymann A, Amanov L, Beltsios E, Arjomandi Rad A, Szczechowicz M, Merzah AS, et al. Minimally invasive direct coronary artery bypass grafting: sixteen years of single-center experience. J Clin Med. 2024;13(11):3338. 38893048 10.3390/jcm13113338 PMC11173276

[13] Sharaf M, Zittermann A, Sunavsky J, Gilis-Januszewski T, Rojas SV, Gotte J, et al. Early and late outcomes after minimally invasive direct coronary artery bypass vs. full sternotomy off-pump coronary artery bypass grafting. Front Cardiovasc Med. 2024;11:1298466. 38450373 10.3389/fcvm.2024.1298466 PMC10914960

[14] Manuel L, Fong LS, Betts K, Bassin L, Wolfenden H. LIMA to LAD grafting returns patient survival to age-matched population: 20-year outcomes of MIDCAB surgery. Interact Cardiovasc Thorac Surg. 2022;35(4): ivac243. 36130278 10.1093/icvts/ivac243 PMC9519092

[15] Gianoli M, de Jong AR, Jacob KA, Namba HF, van der Kaaij NP, van der Harst P, et al. Minimally invasive surgery or stenting for left anterior descending artery disease—meta-analysis. Int J Cardiol Heart Vasc. 2022;40:101046. 35573649 10.1016/j.ijcha.2022.101046 PMC9098394

[16] Naito S, Demal TJ, Sill B, Reichenspurner H, Onorati F, Gatti G, et al. Impact of surgeon experience and centre volume on outcome after off-pump coronary artery bypass surgery: results from the European Multicenter Study on coronary artery bypass grafting (E-CABG) registry. Heart Lung Circ. 2023;32(3):387–94. 36566143 10.1016/j.hlc.2022.11.009

[17] Masroor M, Chen C, Zhou K, Fu X, Khan UZ, Zhao Y. Minimally invasive left internal mammary artery harvesting techniques during the learning curve are safe and achieve similar results as conventional LIMA harvesting techniques. J Cardiothorac Surg. 2022;17(1):203. 36002863 10.1186/s13019-022-01961-0 PMC9404583

[18] Akca F, Ter Woorst J. Learning curve of thoracoscopic nonrobotic harvest of the left internal mammary artery in minimally invasive coronary artery bypass grafting. Innovations. 2023;18(3):262–5. 37294049 10.1177/15569845231178012

[19] Lawton JS, Tamis-Holland JE, Bangalore S, Bates ER, Beckie TM, Bischoff JM, et al. 2021 ACC/AHA/SCAI Guideline for Coronary Artery Revascularization: a report of the American College of Cardiology/American Heart Association Joint Committee on Clinical Practice Guidelines. Circulation. 2022;145(3):e18–114. 34882435 10.1161/CIR.0000000000001038

[20] Thielmann M, Bonaros N, Barbato E, Barili F, Folliguet T, Friedrich G, et al. Hybrid coronary revascularization: position paper of the European Society of Cardiology Working Group on Cardiovascular Surgery and European Association of Percutaneous Cardiovascular Interventions. Eur J Cardiothorac Surg. 2024;66(2): ezae271. 39142801 10.1093/ejcts/ezae271

