# Supplementary figures and images for: Experience in setting up non-robotic minimally invasive direct coronary artery bypass grafting in a non-routine off-pump coronary artery bypass center
Source: Eur J Med Res. 2025 Jan 31;30:64. doi: 10.1186/s40001-025-02320-0 (PMC11783730; doi:10.1186/s40001-025-02320-0)

Development of MIDCAB (n=72)

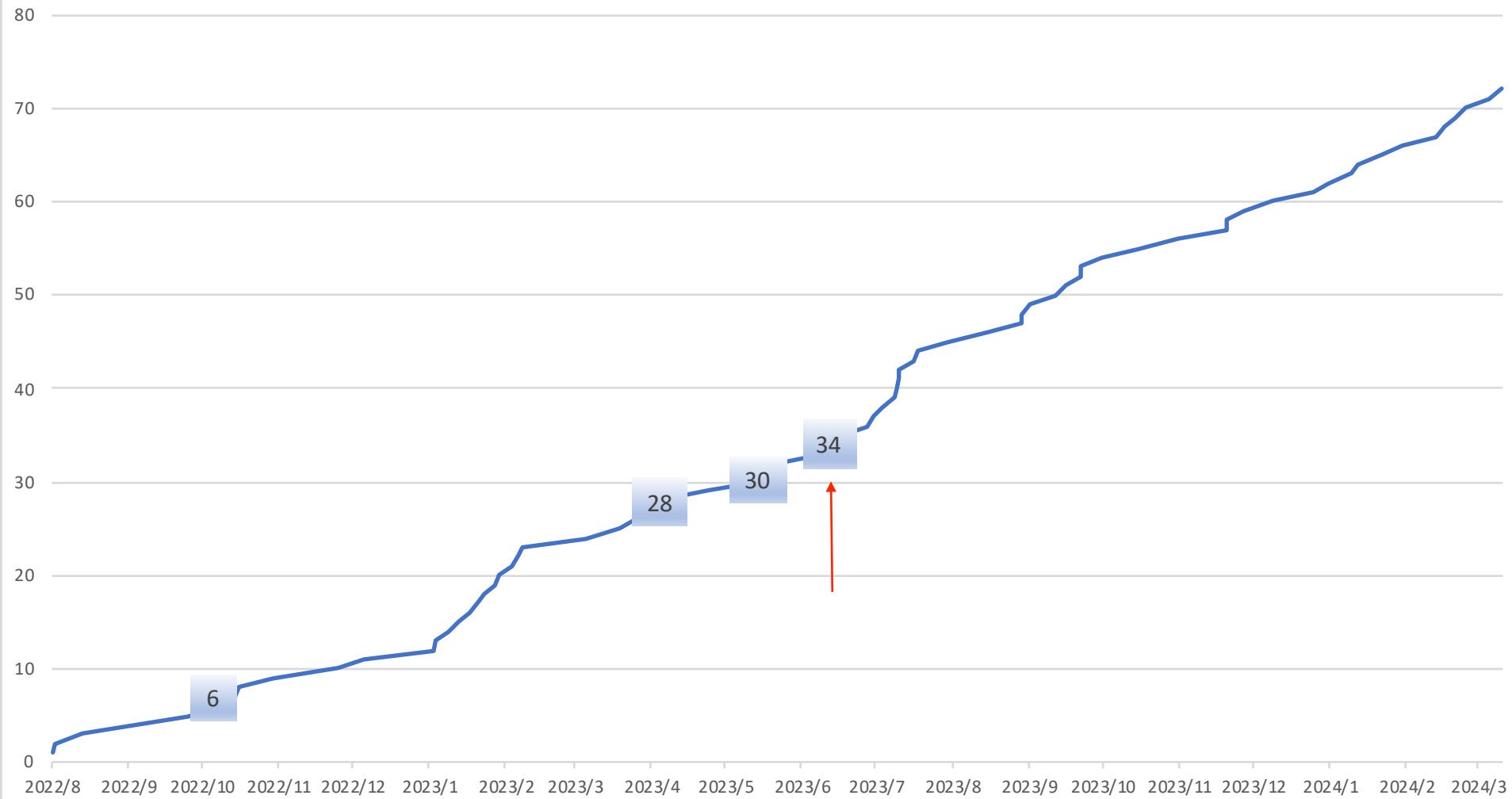

Supplement: Supplementary file 1 — Supplementary Material 1: Suppl. Figure 1. The graphical explanation of the development of MIDCAB patients and the occurrence of MACCE. The number of MIDCAB patients has gradually increased. MACCE occurred in 4 patients with the last MACCE on the 34th patient. MACCE, major adverse cardiac and cerebrovascular event; MIDCAB, minimally invasive direct coronary artery bypass. [file 40001_2025_2320_MOESM1_ESM.pdf]
