# Supplementary material for: Experience in setting up non-robotic minimally invasive direct coronary artery bypass grafting in a non-routine off-pump coronary artery bypass center
Source: Eur J Med Res. 2025 Jan 31;30:64. doi: 10.1186/s40001-025-02320-0 (PMC11783730; doi:10.1186/s40001-025-02320-0)
Supplement: Supplementary file 2 — Supplementary Material 2: Suppl. Table 1. Representative information in MIDCAB patients as a hybrid procedure. [file 40001_2025_2320_MOESM2_ESM.docx]

| **Suppl. Table 1. Representative information in MIDCAB patients as a hybrid procedure** | | | |
| --- | --- | --- | --- |
|  |  | **with PCI (n=18)** | **with TAVI (n=3)** |
| Age (y) | | 70.3±11.6 | 72.3±12.2 |
| Male, n (%) | | 16 (88.9) | 3 (100.0) |
| Euro Score II | | 1.3 (0.7-4.7) | 1.2 (0.7-2.4) |
| LVEF (%) | | 49.9±9.7 | 58.0±3.5 |
| Reason for hybrid procedures | impaired clinical status | 8 (44.4) | 2 (66.7) |
|  | other smaller diameter target vessel | 3 (16.7) | 0 (0.0) |
|  | CVD and low LVEF | 1 (5.6) | 0 (0.0) |
|  | mental disorder | 1 (5.6) | 0 (0.0) |
|  | porcelain aorta | 0 (0.0) | 1 (33.3) |
|  | other | 5 (27.8) | 0 (0.0) |
| Data documented as n (%) or mean ± standard deviation or median (1. quartile-3. quartile). CVD, cerebrovascular disease; LVEF, left ventricular ejection fraction; MIDCAB, minimally invasive direct coronary artery bypass grafting; PCI, percutaneous coronary intervention; TAVI, transcatheter aortic valve implantation | | | |
|  |  |  |  |
|  |  |  |  |
